# Supplementary material for: Lung and Heart Biology of the Dp16 Mouse Model of down Syndrome: Implications for Studying Cardiopulmonary Disease
Source: Genes (Basel). 2023 Sep 19;14(9):1819. doi: 10.3390/genes14091819 (PMC10530394; doi:10.3390/genes14091819)

Supplemental Table S1

Antibody	Vendor	Catalog #	Concentration	Species	Application
IFNAR1	Abclonal	A18594	1:1000	Rabbit	Immunoblot
IFNGR2	Abclonal	A14221	1:1000	Rabbit	Immunoblot
Stat1	Cell Signaling	D4Y6Z	1:1000	Rabbit	Immunoblot
p-Stat1	Invitrogen	33-3400	1:1000	Mouse	Immunoblot
Stat3	Cell Signaling	79D7	1:2000	Rabbit	Immunoblot
pStat3	Cell Signaling	D3A7	1:500	Rabbit	Immunoblot
PAFR	Bioss	1478R	1:500	Rabbit	Immunoblot
IL-10Rb	Bioss	2602R	1:500	Rabbit	Immunoblot
GAPDH	Cell Signaling	D16H11	1:2000	Rabbit	Immunoblot
Anti-rabbit IgG HRP	Cell signaling	7074S	1:2000	Goat	Immunoblot
Anti-mouse IgG HRP	Millipore	AP124P	1:5000	Goat	Immunoblot
CD3	Abcam	AB5690	1:250	Rabbit	Tissue IF
CD45R	Invitrogen	RA3-6B2	1:500	Rat	Tissue IF
Anti-Rat 488	Invitrogen	A4826TR	1:500	Goat	Tissue IF
Anti-Rabbit 594	Invitrogen	A11037	1:500	Goat	Tissue IF

Supplemental Figure S1

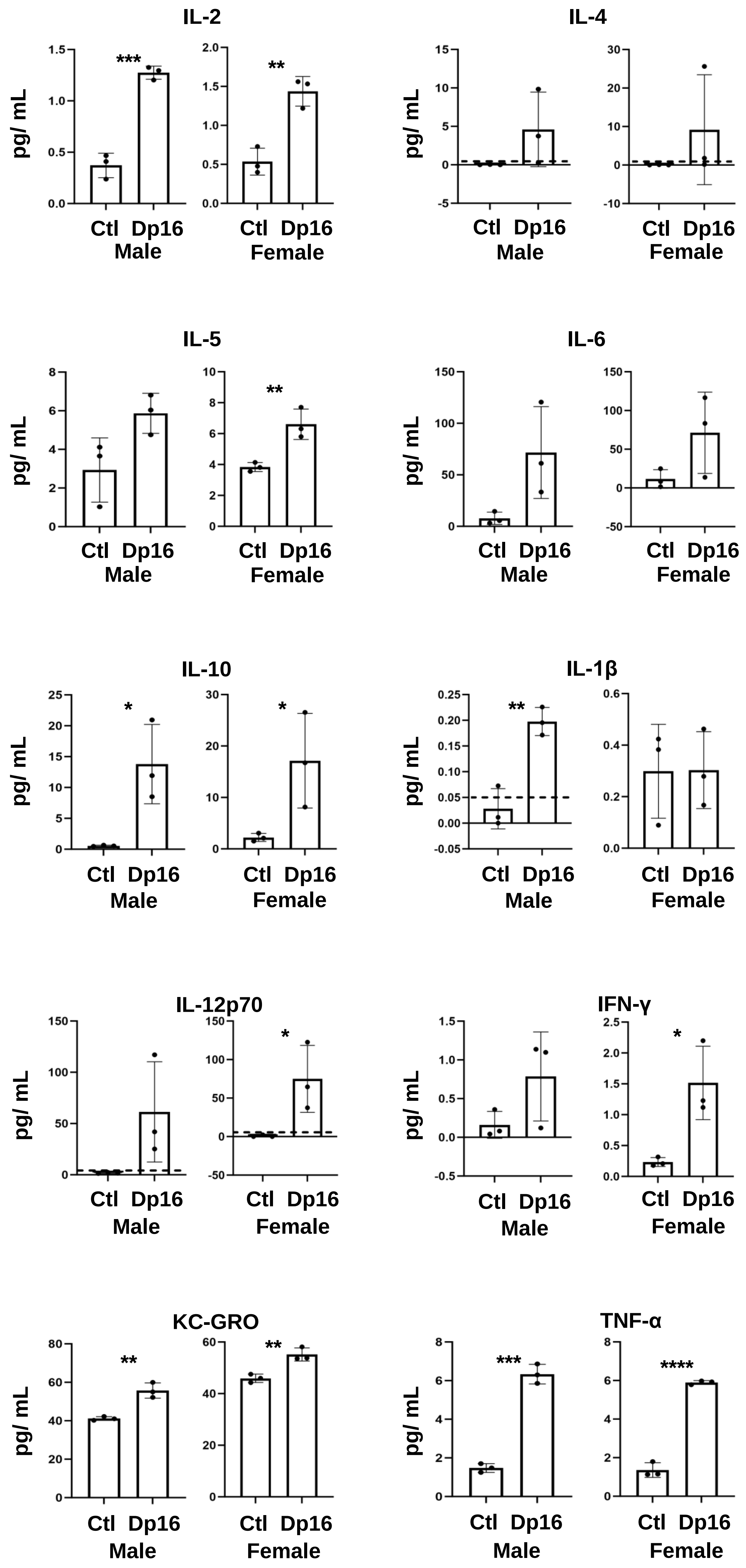

Supplemental Figure S2

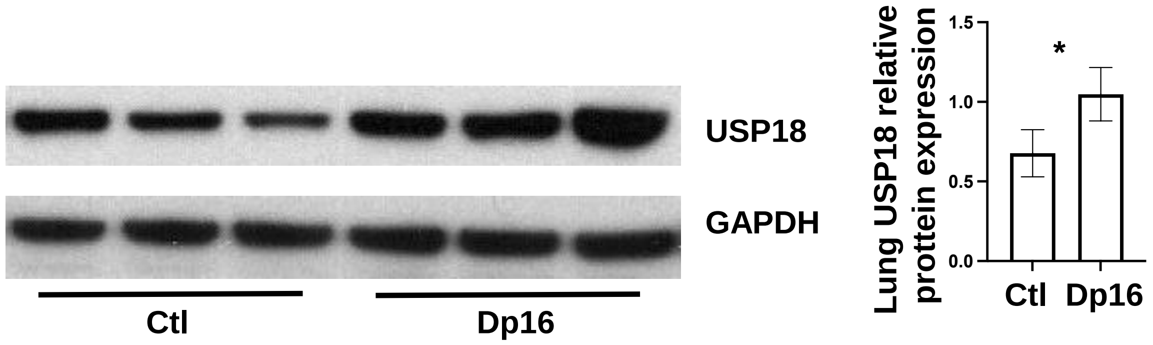

Supplemental Figure S3

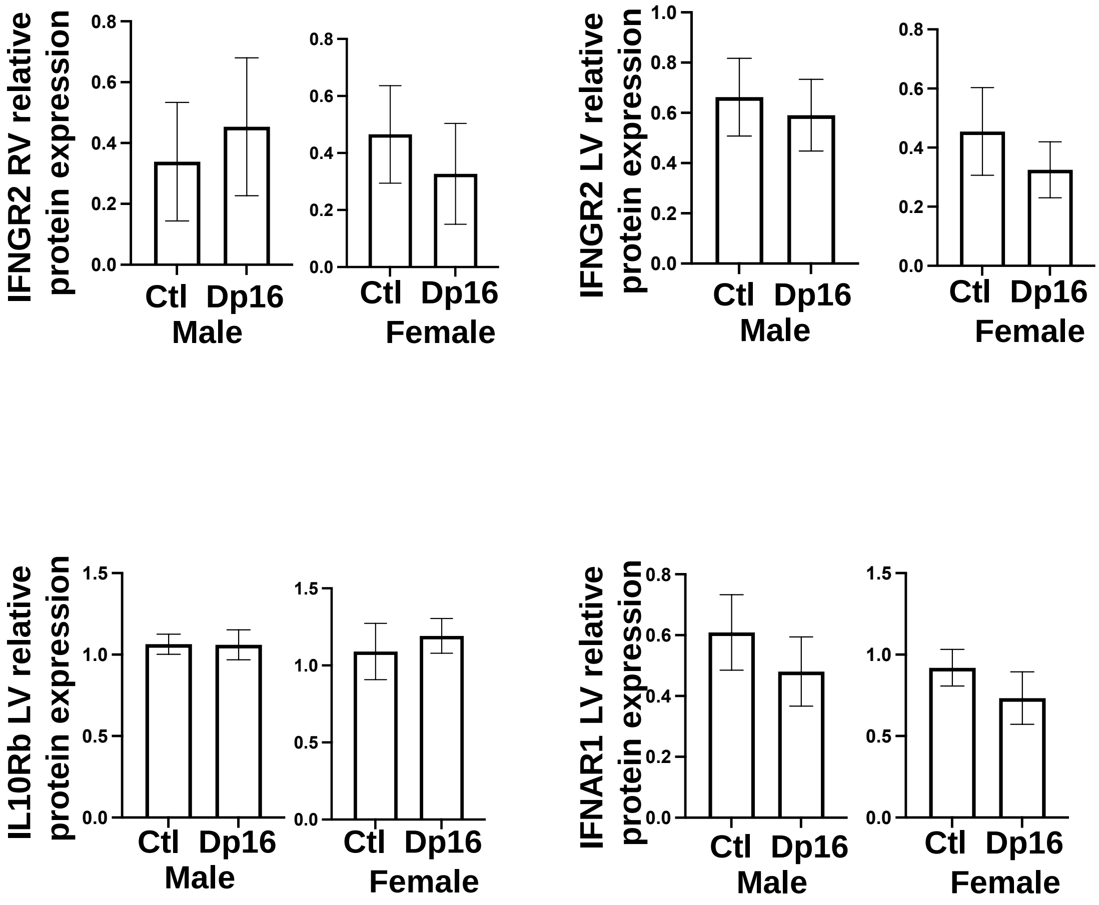

Supplement: Supplementary file 1 [file genes-14-01819-s001.zip › genes-2494334-supplementary.pdf]
